# Degree of Hydration, Microstructure, and Mechanical Properties of Cement-Modified TiO_2_ Nanoparticles

**DOI:** 10.3390/ma17184541

**Published:** 2024-09-15

**Authors:** Young-Cheol Choi

**Affiliations:** Department of Civil and Environmental Engineering, Gachon University, Seongnam 13120, Republic of Korea; zerofe@gachon.ac.kr; Tel.: +82-31-750-5721

**Keywords:** compressive strength, hydration degree, nucleation site, setting time, TiO_2_ nanoparticles

## Abstract

This study investigated the effects of TiO_2_ nanoparticles (TNPs) on the hydration and microstructure of cement. The primary experimental variable was the TNP content, which ranged from 0 to 10 wt% of the cement. Cement paste and mortar specimens incorporating TNPs were prepared to assess the hydration characteristics, microstructure, and mechanical properties of the cement composites. Hydration characteristics were evaluated using heat of hydration, setting time, and thermogravimetric (TG) analysis. The microstructure was assessed through mercury intrusion porosimetry (MIP). The results indicated that TNPs accelerated the hydration of cement and modified the matrix microstructure, decreasing porosity and improving early-age strength. However, their tendency to agglomerate makes proper dispersion crucial for their effective application in construction materials. Therefore, when developing new building materials incorporating TNPs, it is essential to consider the properties of the nanoparticles and their physical and chemical effects on cement.

## 1. Introduction

Cement, and the concrete derived from it, is a widely utilized building material, valued for its high compressive strength and cost-effectiveness compared to other materials. However, advancements in construction technology have revealed problems such as low tensile strength and limited functionality. Recently, a wide range of studies have been conducted to address the limitations of traditional cement and concrete by leveraging the unique properties and advantages of nanoscale materials, such as their inherent characteristics and particle size benefits [1,2,3,4]. TiO_2_ nanoparticles (TNPs) are widely used in various products, such as ultraviolet protection materials, photocatalysts, self-cleaning glass, sunscreen, coatings, and inks, because of their high chemical stability and superhydrophilicity. The application of TNPs in construction materials has garnered considerable attention over the past 20 years because of their effectiveness in adding novel functionalities and performance improvement [5,6]. Specifically, TNPs have been commonly employed to improve the mechanical performance of concrete, including compressive and flexural strength [7,8]. Mustafa et al. studied the shear behavior of reinforced concrete beams using TNPs and silica fume. They reported that TNPs contribute to improvement in both compressive and tensile strength in concrete, as well as an increase in shear strength [9]. Zhang et al. investigated the effects of TNPs on cement hydration and drying shrinkage [10]. Their findings indicated that TNPs enhance the compressive strength of cement and reduce water loss in cementitious materials during the drying process, thereby mitigating drying shrinkage. However, other studies have reported conflicting results. In one study, the compressive strength at 28 days of samples containing 2% TNPs by weight of cement was found to be similar to that of the control samples [11], and in some cases, the addition of TNPs even led to a reduction in compressive strength compared to the control [12]. Cementitious materials are well suited for incorporating TNPs because of their favorable binding characteristics and fine pore structures within the matrix, rendering them ideal for the development of functional construction products.

TNPs can enhance the compressive strength, impact resistance, and tensile strength of concrete [13,14] while also influencing the initial hydration reaction of cement [15,16] and improving performance aspects such as durability [17] and photocatalytic functionality [18,19]. These improvements are typically attributed to the unique properties of TNPs and the seeding and filling effects. TNPs function as a nucleation site, providing more space for the precipitation of cement hydrates, which accelerates early hydration, shortens the induction period, and increases the quantity of hydration products [20,21]. Furthermore, TNPs, being significantly smaller than cement particles, occupy the empty pores between cement clinkers. This helps to reduce pore size, thereby densifying the microstructure of the matrix and enhancing its strength [22]. Unlike nanosilica powder, which generates the additional CSH gel by the pozzolanic reaction [23], TiO_2_ is an inert material that is not reactive with cement. Rahim et al. studied the effect of TNP incorporation on the mechanical properties of concrete. They reported a 42% and 34% improvement in flexural and tensile strength, respectively, when 4% TNPs were used [24]. Li et al. investigated the mechanical properties of cement mortar using various TNP sizes [8]. Their findings indicated that the use of smaller TNPs considerably enhanced strength. Specifically, when the average diameter of the TNPs was 10 nm, the compressive strength of the cement composite improved by approximately 11% compared to when the diameter was 15 nm. This phenomenon occurred because the smaller TNPs provided more sites for nucleation, promoting cement hydration. Essawy et al. revealed that adding 5% TNPs by cement weight resulted in a compressive strength increase of approximately 5% [25]. Quantitative analyses of the influence of TNPs on the hydration reaction, including the resulting changes in matrix microstructure and mechanical properties, as well as comprehensive studies of the roles of TNPs on the initial cement hydration, have been performed to a very limited extent.

This study investigated the effects of TNPs on cement hydration, with a particular focus on the resulting changes in the microstructure of the cement matrix and the corresponding effects on mechanical properties through experimental analyses. Cement paste and mortar specimens were prepared by varying TNP incorporation rates (0%, 1%, 3%, 5% and 10%) using the weight of cement as the primary variable. The effect on the hydration characteristics was assessed using setting-time measurements and isothermal conduction calorimetry tests. Additionally, thermogravimetric (TG) analysis and mercury intrusion porosimetry (MIP) were conducted to quantitatively assess the degree of cement hydration and its effect on the pore structure of cement matrix.

## 2. Experimental Details

### 2.1. Materials

Ordinary Portland cement (OPC), sourced from Korea S, was utilized as the binder. The chemical oxide composition of the OPC, determined through X-ray fluorescence (XRF) analysis (ZSX Primus II, Rigaku, Tokyo, Japan), is presented in Table 1. The TiO_2_ used is a commercially available product, AEROXIDE P25, obtained from Company E in Germany. The densities of OPC and TNP are 3.13 g/cm^3^ and 4.59 g/cm^3^, respectively [26].

**Figure 1:**
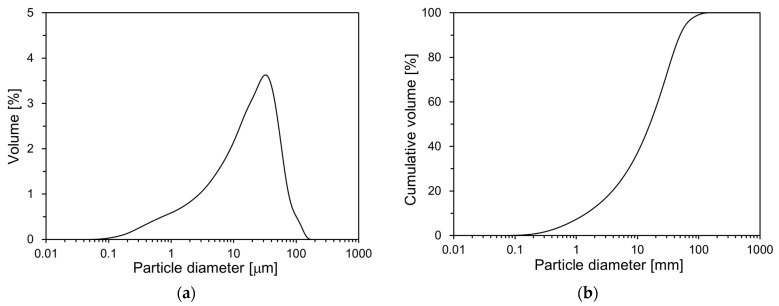
PSD of OPC: (**a**) volume; (**b**) cumulative volume.

Figure 1 illustrates the particle size distribution (PSD) of OPC measured using the laser diffraction method with an LS 230 particle size analyzer (Beckman Coulter, Brea, CA, USA). As depicted in Figure 1, OPC has a particle size range of 0.1 to 150 µm. The average particle size is 20.2 µm.

**Figure 2:**
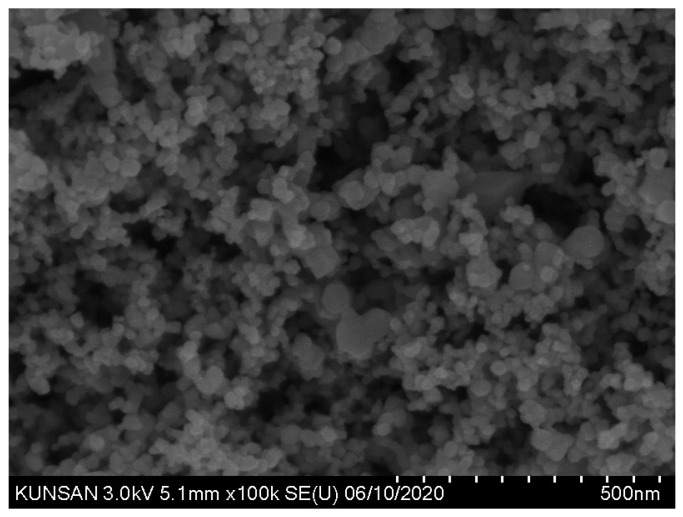
SEM image of P25.

The TNPs, as depicted in Figure 2, comprise nanosized particles that agglomerate. Therefore, the PSD of TNPs measured using the laser diffraction method can be inaccurate [27]. Hence, this study determined the PSD of P25 based on the scanning electron micros-copy (SEM) images displayed in Figure 2.

**Figure 3:**
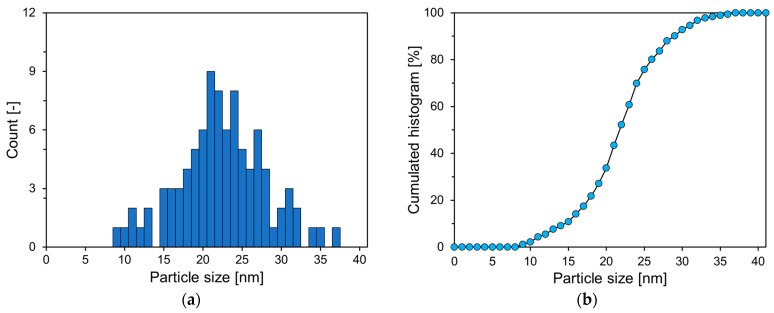
PSD of P25: (**a**) histogram of particle size distribution; (**b**) cumulative histogram.

As presented in Figure 2, P25 predominantly comprises small spherical particles with some larger angular particles. Figure 3 reveals the PSD estimated from SEM image analysis of P25. In the graph in Figure 3, the cumulative histogram of P25 exhibits an S shape, with an average diameter of 22.6 nm.

**Figure 4:**
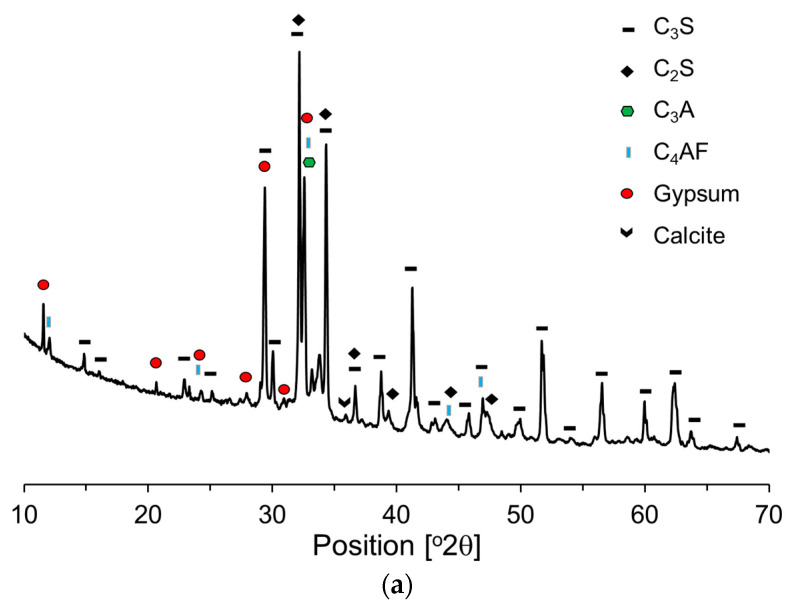

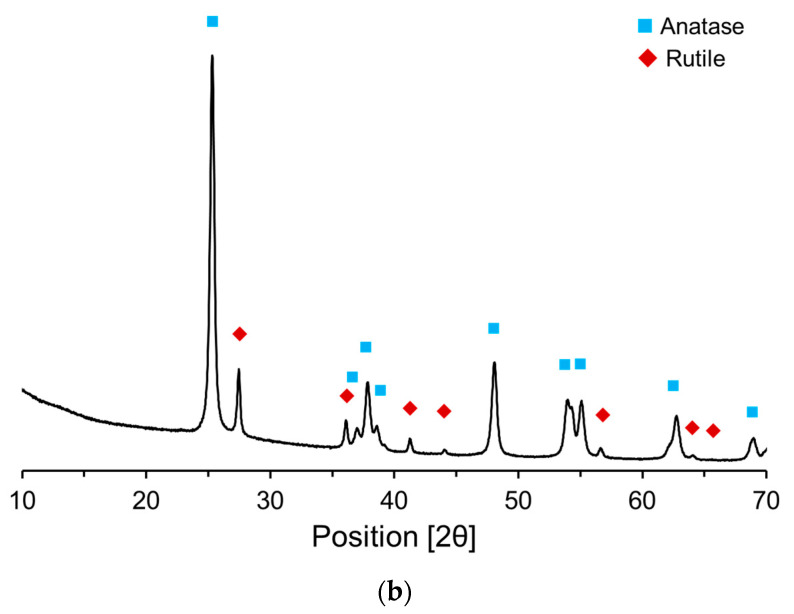
XRD patterns of raw materials: (**a**) OPC; (**b**) P25.

Figure 4 presents the X-ray diffraction (XRD) patterns of the raw materials, offering a comparative analysis of their crystalline structures and phase compositions. Based on these XRD patterns, the primary components of OPC and P25 were quantitatively determined using Rietveld refinement. The primary mineral components of the OPC (Figure 4a) were identified to be C_3_S (58%), C_2_S (16%), C_3_A (7%), and C_4_AF (10%). Additionally, the OPC contained gypsum and limestone powder—2.6% and 4.5%, respectively. The primary crystalline phases of P25 (Figure 4b) are anatase (84.5%) and rutile (15.5%). Generally, anatase and rutile exist either as heterojunction phases or as individual nanosized particles.

### 2.2. Mixture Proportions

In this study, cement paste and mortar specimens incorporating P25 were produced according to Table 2. Additionally, mortar specimens were fabricated to investigate the compressive strength. The primary variable in the study was the incorporation rate of P25, which was tested at 0%, 1%, 3%, 5%, and 10% by weight of cement. The amount of sand in the mortar specimen for compressive strength measurement was three times the weight of the cement. Standard sand (ISO 697) was used, and a polycarboxylate superplasticizer was added as a chemical admixture (SP) at 0.3% of the cement weight.

Due to the high specific surface area of TNPs, their incorporation into cement tends to reduce workability. Therefore, an evaluation was conducted to assess the decrease in workability with varying TNP content. In this study, the workability of the mortar specimens was assessed using a mini slump cone test following the guidelines of ASTM C 143. The slump measurement results are presented in Table 2, indicating a decreasing trend in slump with increasing TNP content. Specifically, the slump showed a sharp decline compared to the plain mixture up to 3% TNP incorporation, after which the decrease became more gradual as the TNP content increased further.

### 2.3. Test Methods

The morphology and particle size of P25 were analyzed using images obtained with a scanning electron microscope (SEM) (Zeiss Sigma 500, Baden-Württemberg, Germany). Compressive strength measurements were conducted at 3, 7, 28, and 91 days in accordance with ISO 679 using mortar specimens. The mineral composition of the raw material was analyzed using a PANalytical X’Pert Pro MPD X-ray diffractometer (Malvern Panalytical Ltd., Malvern, UK). The diffractometer scans were conducted over a 2θ range of 10° to 70° with a step size of 0.04°. To quantitatively analyze the effect of P25 on the hydration degree of cement, thermogravimetric (TG) analysis was performed on the cement paste at different ages. The TG analysis was performed using an SDT Q600 (TA instruments, Newcastle, DE, USA) apparatus and measured in a nitrogen atmosphere up to a maximum temperature of 1000 °C. MIP to investigate changes in the micropore structure due to P25 incorporation. The pore size distribution and microstructure change in specimens was estimated using an AutoPore IV 9500 (Micromeritics, Norcross, GA, USA). The paste specimens prepared for the MIP measurements were crushed into pieces approximately 1–2 mm in size. The sample for the MIP measurements was obtained from the middle part of the specimen. The crushed samples were vacuum-dried to halt hydration.

## 3. Results and Discussion

### 3.1. Hydration Characteristics

**Figure 5:**
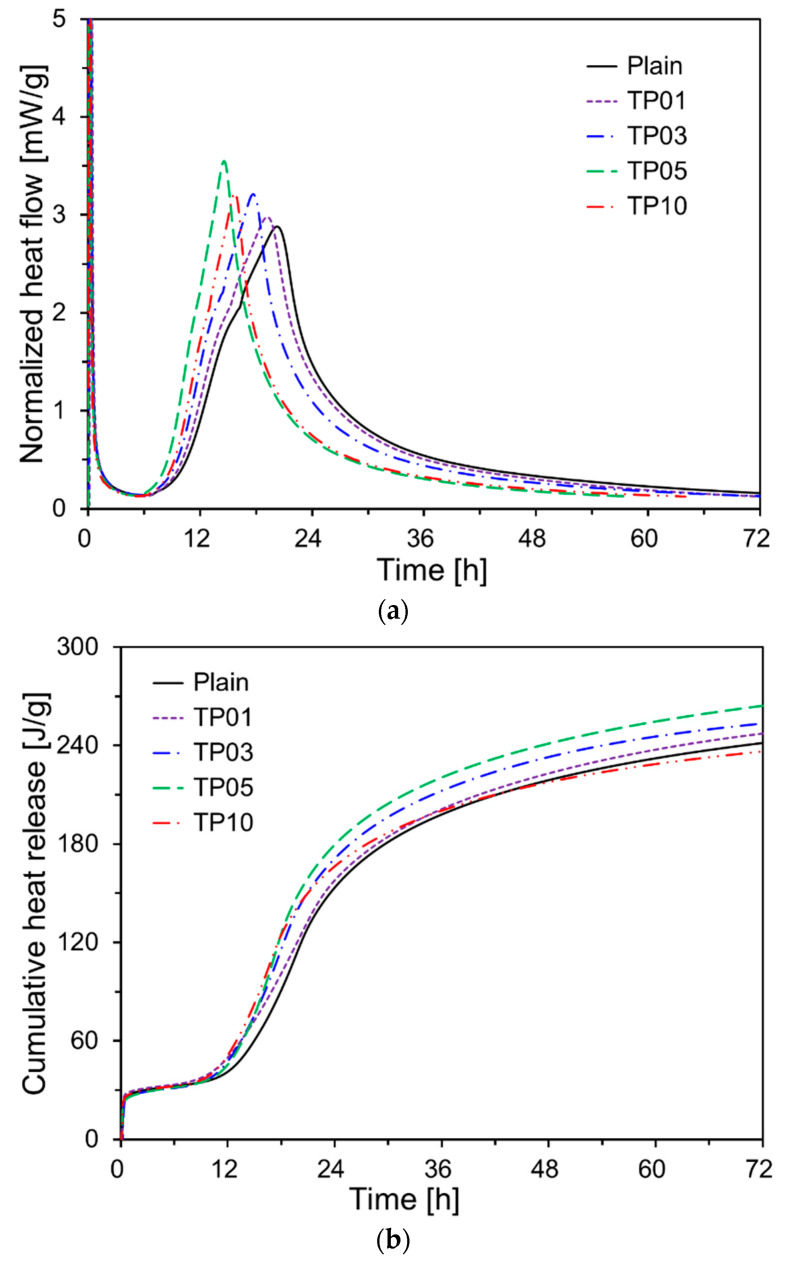
Heat of hydration results of specimens: (**a**) normalized heat flow; (**b**) cumulative heat release.

The normalized heat flow and cumulative heat release of cement paste with 0%, 1%, 3%, 5%, and 10% of P25 by weight are shown in Figure 5. As presented in Figure 5a, the second peak occurred earlier in all the samples with P25 than in Plain. When P25 was increased to 5% of the cement weight, the occurrence time of the second peak tended to decrease, while the peak intensity increased. This effect is likely due to the TNPs accelerating the cement hydration, particularly C_3_S. The seeding effect of TNPs accelerated cement hydration, resulting in a reduced the induction period [21]. Additionally, the microstructure became denser due to the filling effect of TNPs filling the spaces between the cement particles. Similar effects have been reported for cement pastes incorporating nanosilica powder [28].

However, when 10% P25 by weight of cement was added (TP10), cement hydration was not promoted as effectively as in TP5: instead, it tended to be delayed. This is likely because of TNP agglomeration. In the cement paste, the agglomeration of TNPs involves individual nanosized particles clustering to form larger aggregates. The degree of agglomeration depends on various factors, including the surface charge, particle size, concentration, pH of the medium, ionic strength, and the presence of surfactants or polymers. The agglomeration of TNPs negatively affects the homogeneity of the nanoparticle distribution, reducing the exposed surface area of TNPs and consequently decreasing its photocatalytic efficiency [29]. Furthermore, the uneven distribution of nanoparticles is unlikely to positively impact the mechanical and durability performance of concrete structures [30].

Figure 5b presents the cumulative heat release curves for all specimens. At 42 h into the hydration reaction, the cumulative heat release in most samples containing P25 was higher than that of Plain. However, the cumulative heat release of TP10 expressed at 72 h was 95% of that of Plain. By contrast, the 72 h cumulative heat releases for TP1, TP3, and TP5 were 100.8%, 102.9%, and 107.2%, respectively, relative to that of Plain.

By contrast, the cumulative heat releases at 72 h for TP1, TP3, and TP5 were 100.8%, 102.9%, and 107.2%, respectively, relative to that of Plain. The samples with P25 exhibited higher early hydration reactions than those of Plain after 10 h. A substantial body of research has indicated that not only reactive fine particles but also non-reactive particles can accelerate the cement hydration process through the filler effect [31,32,33].

TNPs contribute to the early hydration process of cement by acting as nucleation sites for the precipitation of hydration products, specifically C-S-H gel, between cement particles. These particles preferentially adsorb the C-S-H gel formed during hydration, and the adsorbed gel propagates between the cement particles, serving as nucleation centers. This mechanism facilitates hydration by forming a connected C-S-H network, thereby improving the mechanical properties of the cement system [34,35,36].

### 3.2. Setting Behavior of Specimens

**Figure 6:**
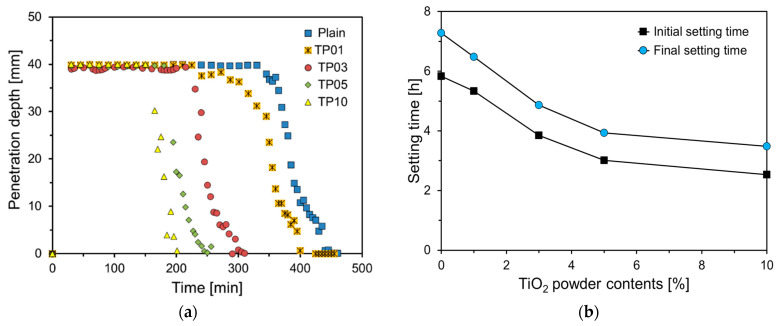
Penetration depth and setting times of specimens: (**a**) penetration depth; (**b**) setting time.

The setting behavior of samples was measured using the results of Vican needle penetration depth (ISO 9597). The graph of penetration depth in Figure 6a indicates that as the amount of P25 increases, the penetration depth curves shift to the left. Materials with a high specific surface area, such as P25, have a greater capacity to adsorb water, resulting in the rapid consumption of free water within the matrix. This accelerates the bridging of the gaps within the paste, thereby increasing its viscosity and promoting setting [22].

Figure 6b depicts the graph of setting times of specimens. For the Plain specimen, the initial setting time was 5.83 h and the final setting time was 7.28 h. The initial setting times for TP01, TP03, TP05, and TP10 were 91.4%, 66.0%, 51.7%, and 43.4%, respectively, that of Plain. Up to a 5% incorporation rate of P25, the initial setting time decreased almost linearly and sharply with the increase in the P25 content, whereas at a 10% incorporation rate, the decrease was gradual. The final setting times for TP01, TP03, TP05, and TP10 were 6.48, 4.87, 3.93, and 3.48 h, respectively, exhibiting a similarly decreasing trend as the amount of P25 incorporated increased, as observed in the initial setting time results.

### 3.3. Compressive Strength

**Figure 7:**
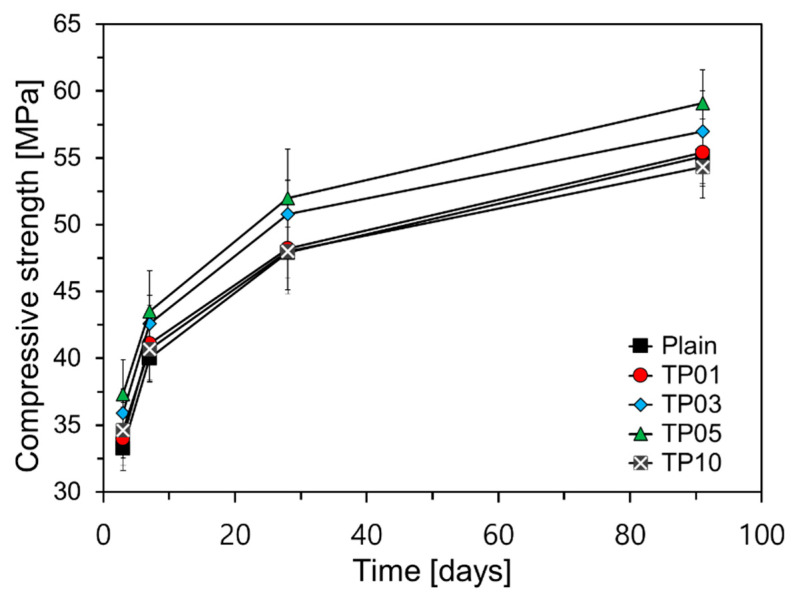
Graph of compressive strength evolution over curing time.

Figure 7 illustrates the results of compressive strength over curing age. As shown in Figure 7, a significant strength improvement was observed for P25 content up to 5%. The observed increase in compressive strength can be attributed to the accelerated hydration of cement facilitated by fine particles like P25, as discussed in Section 3.2. However, TP10, containing 10% P25, did not exhibit a substantial strength increase compared to TP05, with its 28-day compressive strength being comparable to that of TP01. Moreover, its 91-day compressive strength was 0.8 MPa lower than that of Plain.

**Figure 8:**
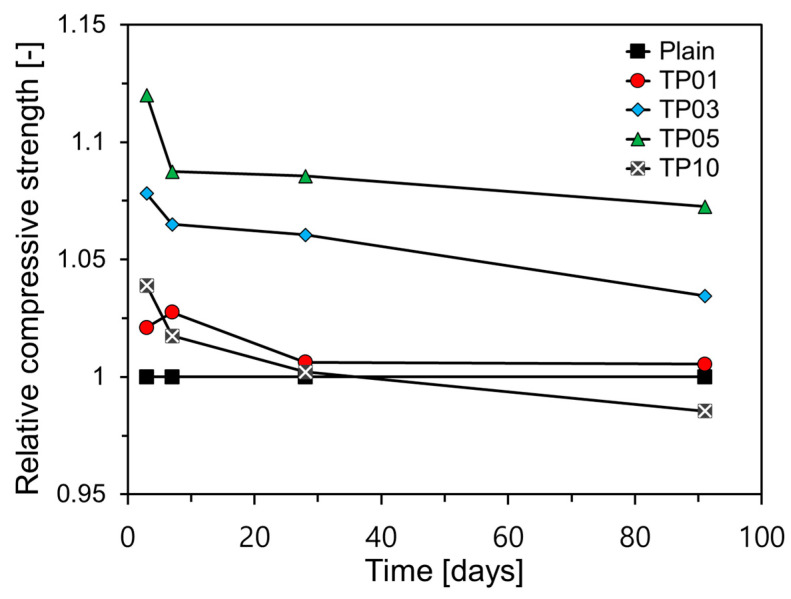
Relative compressive strengths over age.

Figure 8 presents a comparison of the relative compressive strength (RCC) by age for each variable. In this study, relative compressive strength is defined as shown in Equation (1).

(1) $$RCC=\frac{f_{t}}{f_{P}}$$

where $f_{t}$ and $f_{P}$ represent the compressive strength of the target sample and Plain, respectively.

The RCC of all samples decreased as the age increased. With the exception of TP1, all the samples containing P25 exhibited the highest RCC after three days. This strength sharply declined by day 7 and continued to gradually decrease until day 91. This pattern is attributed to the significant impact of TNPs on the early-age hydration of C_3_A and C_3_S, whereas their effect on the hydration of C_2_S, which has a slow reaction and influences long-term properties, is minimal [22].

Nanosilica powder has been shown to accelerate the early hydration of cement, thereby enhancing its early-age mechanical properties [37,38,39,40,41]. In addition, because of its high surface reactivity, nanosilica powder promotes a pozzolanic reaction [38,41,42]. TNPs exhibit similar effects to nanosilica powder in the reaction of cement hydration, enhancing early-age strengths and durability of cement matrices by filling both micropores and nanopores [43,44]. However, unlike nanosilica, TNPs are not pozzolanic.

### 3.4. Pore Structure

The change in pore structure of specimens was measured using mercury intrusion porosimetry (MIP). The porosity measurements for all specimens are presented in Table 3. As shown in Table 3, overall, the addition of P25 tended to reduce the porosity of the cement paste. As the P25 content increased to 5% by weight of cement, the porosity decreased accordingly. However, when the P25 content reached 10%, porosity increased again and returned to a level similar to that of TP01.

**Figure 9:**
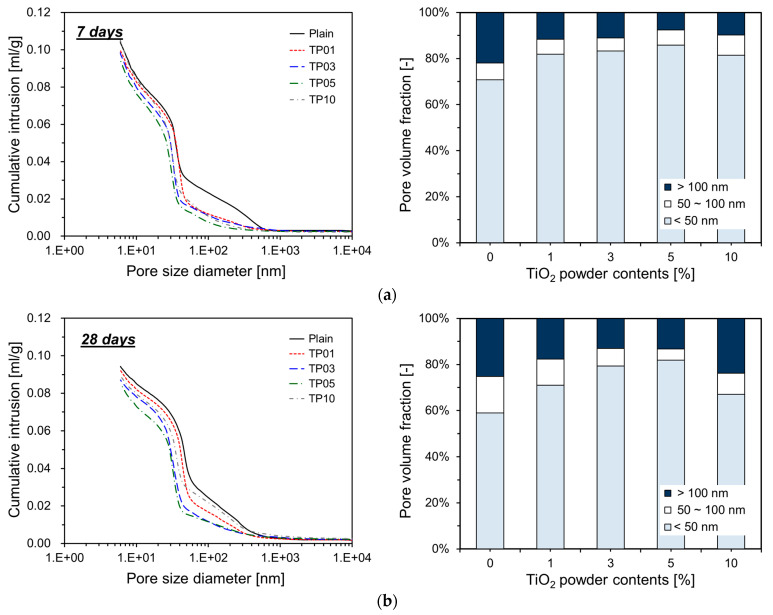

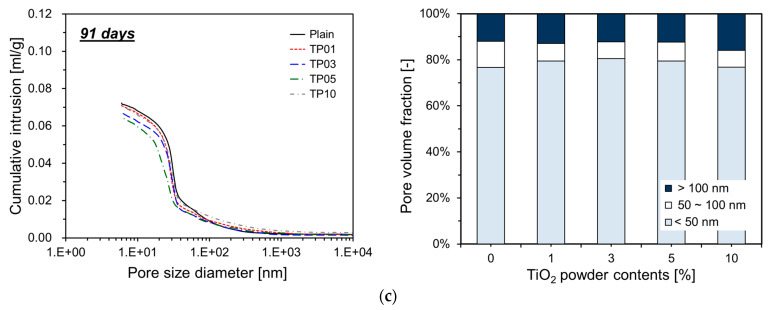
Cumulative intrusion and pore volume fraction of cement paste containing P25: (**a**) 7 days; (**b**) 28 days; (**c**) 91 days.

Figure 9 details the measurement results obtained by MIP. As shown, the incorporation of P25 modifies the cumulative intrusion of specimens. For all ages, the cumulative intrusion of the specimens with P25 is lower than that of Plain. When P25 was added up to 5%, the cumulative intrusion decreased proportionally as the P25 content increased. This phenomenon implies that the TNPs function effectively as fillers in the pore spaces.

As the hydration reaction of the cement progresses, aggregates incorporating nanoparticles as nucleation sites expand and gradually occupy the surrounding pore spaces. These nucleation sites considerably accelerate the cement hydration reaction, resulting in hydrates that rapidly fill the pores. Consequently, the porosity is reduced when P25 is incorporated into cement. This effect is more pronounced at 7 and 28 days, but less so at 91 days. Figure 9 illustrates that at 7 and 28 days, the specimens with P25 exhibit an increased proportion of capillary pores smaller than 50 nm within the total pore volume. This phenomenon implies that the TNPs function as nucleation sites, promoting the generation of additional hydrates that occupy the pores in the cement matrix, reducing their size [22]. However, at 91 days, the influence of P25 incorporation on the pore volume fraction was less pronounced than that at earlier ages. This result could be attributed to the inert properties of the TNPs, which likely accelerated the substantial generation of hydrates by day 28, resulting in the filling of most capillary pores. Therefore, further growth of hydration products could have been restricted by the availability of space. For TP10, which included 10% P25, the pore volume increased compared with TP05, resulting in a pore distribution similar to TP01 because P25 particles tend to agglomerate easily in aqueous solutions. When a large quantity of P25 was added, agglomeration hindered the particles from effectively serving as nucleation sites.

### 3.5. TG Analysis

**Figure 10:**
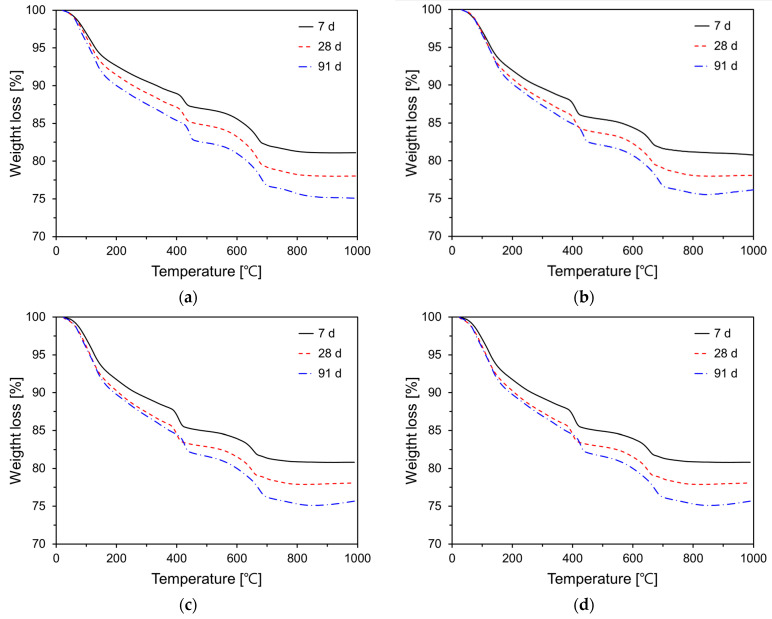
TG curves of specimens containing P25 at 7, 28, and 91 days: (**a**) Plain; (**b**) TP03; (**c**) TP05; (**d**) TP10.

The degree of cement hydration is a quantitative indicator that reflects the extent of the reaction of cement clinker particles within the matrix, and it is influenced by various factors such as supplementary cementitious materials, curing temperature, and age. This degree of cement hydration significantly affects the physical and chemical properties of cement or concrete. Typically, techniques such as calorimetry, thermogravimetric analysis (TG), and scanning electron microscopy are employed to quantify the degree of hydration. In this study, the degree of cement hydration was quantified by calculating the chemically bound water using the TG results. Figure 10 illustrates the TG analysis results for various P25 incorporation rates (0%, 3%, 5%, and 10%). When the cement paste specimens are exposed to elevated temperatures, weight loss occurs within a specific temperature range due to the dehydration, decomposition, and decarbonation of hydration products [45,46]. The TG graphs of all variables were similar in shape. This phenomenon indicates that the types of hydration products are similar. The TG curve of cement paste is typically delineated into the following regions: 40–200 °C region, where dehydration of calcium silicate hydrate gels and calcium aluminate hydrates occur (w_b1_); 400–500 °C region, where the decomposition of portlandite occurs (w_b2_); and 500–750 °C region, where the decarbonation of CaCO_3_ occurs (w_b3_) [47,48].

The chemically bound water content (w_b_) was quantified by measuring the relative weight loss observed in the three aforementioned temperature ranges, as expressed in Equation (2). The coefficient 0.41 in Equation (2) is derived from carbonated portlandite and is used to estimate the w_b_ [49,50]. The degree of cement hydration (α) is defined by Equation (3). The coefficient 0.24 in Equation (3) denotes the maximum content of w_b_ required to fully hydrate cement 1 g, which generally falls within the range 0.23–0.25 g [49,50].

(2) $$w_{b}=w_{b1}+w_{b2}+0.41\times w_{b3}$$

(3) $$\alpha =\frac{w_{b}}{0.24}\times 100$$

**Figure 11:**
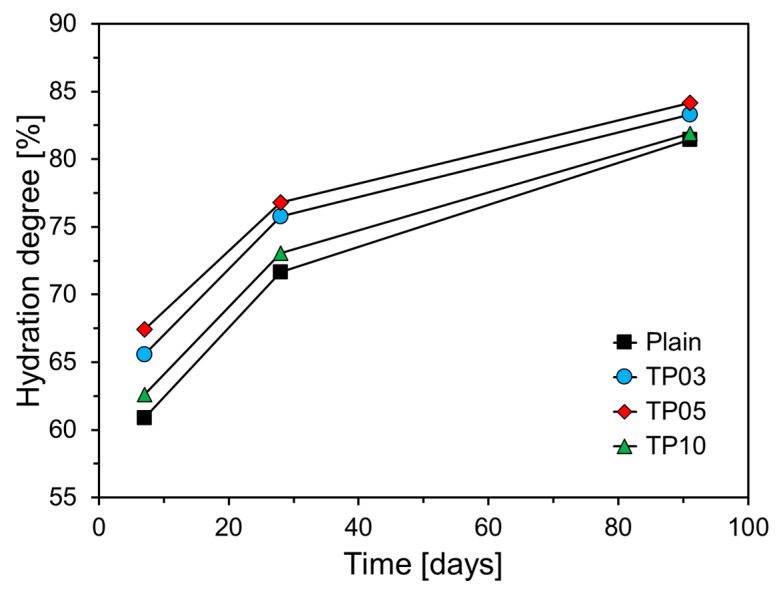
Hydration degree of specimens at various curing ages.

Figure 11 depicts the degree of cement hydration with respect to P25 incorporation. All variables revealed an increasing trend in hydration degree as the curing age increased. Specimens with P25 exhibited a higher hydration degree than that of Plain, suggesting that P25 promotes C_3_S hydration. This observation aligns with the results of cumulative heat release and compressive strength. The specimen with 5% P25 by weight of cement (TP05) exhibited the highest hydration degree at all curing ages, notably showing approximately 6.6% higher hydration at 7 days compared with Plain. The hydration reaction in the specimens with P25 was markedly accelerated at early ages, with its influence diminishing at later ages. This phenomenon indicates that TNPs substantially increased the content of w_b_ at early ages, thereby accelerating the generation and growth of hydrates and enhancing the degree of cement hydration.

## 4. Conclusions

Cementitious materials possess strong binding properties that effectively immobilize TNPs within the matrix, facilitating their integration without the need for additional processing. This study conducted an experimental investigation into the effects of TNP incorporation on hydration degree, pore structure, and compressive strength. A detailed quantitative analysis of the impact of TNPs on matrix microstructure and mechanical properties, including their role in cement hydration, was considered particularly valuable. The incorporation of TNPs led to early-age microstructural changes in the cement paste, resulting in improved compressive strength. TNPs functioned as fillers, reducing void spaces, and served as additional nucleation sites, accelerating the hydration reaction. This acceleration promoted the filling of voids with hydration products in the early stages, enhancing both microstructure and mechanical performance. However, when higher amounts of TNPs were added, their impact on hydration, microstructure, and compressive strength did not increase proportionally. This diminished effect is likely due to the tendency of TNPs to agglomerate, which reduces their efficiency as nucleation sites during mixing. Therefore, when developing advanced functional construction materials, such as photocatalytic or self-cleaning products, it is essential to carefully consider the influence of TNPs on the hydration characteristics of the material. Ensuring the proper dispersion of TNPs within the matrix is crucial for maximizing their functional benefits.

## Figures and Tables

**Table 1:** Chemical oxide composition of OPC by XRF (wt%).

	CaO	MgO	Al_2_O_3_	Fe_2_O_3_	SiO_2_	K_2_O	Na_2_O	SO_3_	LOI
OPC	61.6	2.9	4.5	3.6	19.8	1.2	0.3	2.1	1.2

**Table 2:** Mixture proportion of specimens.

Specimens	W/B (-)	OPC (g)	P25 (g)	Sand (g)	SP (wt.% by Binder)	Slump (mm)
Plain	0.5	1000	-	3000	0.3	120
TP01		990	10			88
TP03		970	30			66
TP05		950	50			62
TP10		900	100			60

**Table 3:** Porosities of specimens after 7-, 28- and 91-day curing.

Sample	Porosity (%)				
	Plain	TP1	TP3	TP5	TP10
7 days	20.40	19.57	19.27	18.55	20.61
28 days	18.46	18.07	17.05	17.05	17.48
91 days	14.15	13.90	13.15	13.15	13.89

## Data Availability

Data are contained within this article.

## References

[1] Zhang H., Yang Z., Su Y. (2019). Hydration kinetics of cement-quicklime system at different temperatures. Thermochim. Acta.

[2] Loh K., Gaylarde C.C., Shirakawa M.A. (2018). Photocatalytic activity of ZnO and TiO_2_ ‘nanoparticles’ for use in cement mixes. Constr. Build. Mater..

[3] Liu M., Tan H., He X. (2019). Effects of nano-SiO_2_ on early strength and microstructure of steam-cured high volume fly ash cement system. Constr. Build. Mater..

[4] Xu Z., Ji Y., Zhang J., Zhang Z., Xue Q., Gao F. (2023). Mechanism of nano-SiO_2_ internal generation for modification of cement-based materials. J. Build. Eng..

[5] Ng D.S., Paul S.C., Anggraini V., Kong S.Y., Qureshi T.S., Rodriguez C.R., Liu Q., Šavija B. (2020). Influence of SiO_2_, TiO_2_ and Fe_2_O_3_ nanoparticles on the properties of fly ash blended cement mortars. Constr. Build. Mater..

[6] Llano-Guerrero E.A., Gómez-Zamorano L.Y., Jiménez-Relinque E. (2020). Effect of the addition of TiO_2_ nanoparticles in alkali-activated materials. Constr. Build. Mater..

[7] Duan P., Yan C.J., Luo W.J., Zhou W. (2016). Effects of adding nano-TiO_2_ on compressive strength, drying shrinkage, carbonation and microstructure of fluidized bed fly ash based geopolymer paste. Constr. Build. Mater..

[8] Li Z., Wang J.L., Li Y., Yu X., Han B.G. (2018). Investigating size effect of anatase phase nano-TiO_2_ on the property of cement-based composites. Mater. Res. Express..

[9] Mustafa T.S., El Hariri M.O.R., Nader M.A., Montaser W.M. (2022). Enhanced shear behaviour of reinforced concrete beams containing nano-titanium. Eng. Struct..

[10] Zhang R., Cheng X., Hou P., Ye Z. (2015). Influences of nano-TiO_2_ on the properties of cement-based materials: Hydration and drying shrinkage. Constr. Build. Mater..

[11] Noorvand H., Abang Ali A.A., Demirboga R., Farzadnia N., Noorvand H. (2013). Incorporation of nano TiO_2_ in black rice husk ash mortars. Constr. Build. Mater..

[12] Meng T., Yu Y., Qian X., Zhan S., Qian K. (2012). Effect of nano-TiO_2_ on the mechanical properties of cement mortar. Constr. Build. Mater..

[13] Han B., Wang Y., Dong S., Zhang L., Ding S., Yu X., Ou J. (2015). Smart concretes and structures: A review. J. Intell. Mater. Syst. Struct..

[14] Jiang S., Zhou D., Zhang L., Ouyang J., Yu X., Cui X.X., Han B. (2018). Comparison of compressive strength and electrical resistivity of cementitious composites with different nano- and micro-fillers. Arch. Civ. Mech. Eng..

[15] Ma B.G., Li H.N., Li X.G., Mei J.P., Lv Y. (2016). Influence of nano-TiO_2_ on physical and hydration characteristics of fly ash-cement systems. Constr. Build. Mater..

[16] Francioso V., Moro C., Martinez-Lage I., Velay-Lizancos M. (2019). Curing temperature: A key factor that changes the effect of TiO_2_ nanoparticles on mechanical properties, calcium hydroxide formation and pore structure of cement mortars. Cem. Concr. Compos..

[17] Mohseni E., Miyandehi B.M., Yang J., Yazdi M.A. (2015). Single and combined effects of nano-SiO_2_, nano-Al_2_O_3_ and nano-TiO_2_ on the mechanical, rheological and durability properties of self-compacting mortar containing fly ash. Constr. Build. Mater..

[18] Aïssa A.H., Puzenat E., Plassais A., Herrmann J.M., Haehnel C., Guillard C. (2011). Characterization and photocatalytic performance in air of cementitious materials containing TiO_2_. Case study of formaldehyde removal. Appl. Catal. B.

[19] Yousefi A., Allahverdi A., Hejazi P. (2013). Effective dispersion of nano-TiO_2_ powder for enhancement of photocatalytic properties in cement mixes. Constr. Build. Mater..

[20] Reches Y. (2018). Nanoparticles as concrete additives: Review and perspectives. Constr. Build. Mater..

[21] Guo C., Wang E., Hou X., Chen J., Zhang W., Ye J., Qin S. (2020). Characterization and mechanism of early hydration of calcium aluminate cement with anatase-TiO_2_ nanospheres additive. Constr. Build. Mater..

[22] Chen J., Kou S.C., Poon C.S. (2012). Hydration and properties of nano-TiO_2_ blended cement composites. Cem. Concr. Compos..

[23] Singh L.P., Karade S.R., Bhattacharyya S.K., Yousuf M.M., Ahalawat S. (2013). Beneficial role of nanosilica in cement based materials—A review. Constr. Build. Mater..

[24] Rahim A., Nair R. (2016). Influence of nanomaterials in high strength concrete. J. Chem. Pharm. Sci..

[25] Essawy A.A., El S.A. (2014). Physico-mechanical properties, potent adsorptive and photocatalytic efficacies of sulfate resisting cement blends containing micro silica and nano-TiO_2_. Constr. Build. Mater..

[26] Yoo S.W., Lee J.W., Park B., Choi Y.C. (2022). Photocatalytic NOx degradation performance of TiO_2_-nanofiber-spray-coated foam composite according to saturated conditions. Constr. Build. Mater..

[27] Xu F. (2018). Review of analytical studies on TiO_2_ nanoparticles and particle aggregation, coagulation, flocculation, sedimentation, stabilization. Chemosphere.

[28] Hou P., Kawashima S., Wang K., Corr D.J., Qian J., Shah S.P. (2013). Effects of colloidal nanosilica on rheological and mechanical properties of fly ash-cement mortar. Cem. Concr. Compos..

[29] Pellegrino F., Pellutiè L., Sordello F., Minero C., Ortel E., Hodoroaba V.D., Maurino V. (2017). Influence of agglomeration and aggregation on the photocatalytic activity of TiO_2_ nanoparticles. Appl. Catal. B.

[30] Li V.C. (2019). Multi-functional engineered cementitious composites (ECC). Engineered Cementitious Composites (ECC).

[31] Gutteridge W.A., Dalziel J.A. (1990). Filler cement: The effect of the secondary component on the hydration of Portland cement. Cem. Concr. Res..

[32] Poppe A.M., De Schutter G.D. (2005). Cement hydration in the presence of high filler contents. Cem. Concr. Res..

[33] Kadri E.H., Duval R. (2009). Hydration heat kinetics of concrete with silica fume. Constr. Build. Mater..

[34] Gartner E.M., Young J.F., Damidot D.A., Jawed I., Bensted J., Barnes P. (2002). Hydration of Portland cement. Structure and Performance of Cements.

[35] Lee B.Y., Kurtis K.E. (2010). Influence of TiO_2_ nanoparticles on early C_3_S hydration. J. Am. Ceram. Soc..

[36] Thomas J.J. (2007). A new approach to modeling the nucleation and growth kinetics of tricalcium silicate hydration. J. Am. Ceram. Soc..

[37] Ji T. (2005). Preliminary study on the water permeability and microstructure of concrete incorporating nano-SiO_2_. Cem. Concr. Res..

[38] Björnström J., Martinelli A., Matic A., Börjesson L., Panas I. (2004). Accelerating effects of colloidal nano-silica for beneficial calcium–silicate–hydrate formation in cement. Chem. Phys. Lett..

[39] Ye Q., Zhang Z., Kong D., Chen R. (2007). Influence of nano-SiO_2_ addition on properties of hardened cement paste as compared with silica fume. Constr. Build. Mater..

[40] Senff L., Labrincha J.A., Ferreira V.M., Hotza D., Repette W.L. (2009). Effect of nano-silica on rheology and fresh properties of cement pastes and mortars. Constr. Build. Mater..

[41] Li G. (2004). Properties of high-volume fly ash concrete incorporating nano-SiO_2_. Cem. Concr. Res..

[42] Jo B.W., Kim C.H., Tae G.H., Park J.B. (2007). Characteristics of cement mortar with nano-SiO_2_ particles. Constr. Build. Mater..

[43] Lucas S.S., Ferreira V.M., De Aguiar J.L.B. (2013). Incorporation of titanium dioxide nanoparticles in mortars—Influence of microstructure in the hardened state properties and photocatalytic activity. Cem. Concr. Res..

[44] Shafaei D., Yang S., Berlouis L., Minto J. (2020). Multiscale pore structure analysis of nano titanium dioxide cement mortar composite. Mater. Today Commun..

[45] Yu R., Spiesz P., Brouwers H.J.H. (2014). Effect of nano silica on the hydration and microstructure development of ultra-high performance concrete (UHPC) with a low binder amount. Constr. Build. Mater..

[46] Chen L., Lin D.F. (2009). Application of sewage sludge ash and nano-SiO_2_ to manufacture tile as construction material. Constr. Build. Mater..

[47] Pane I., Hansen W. (2005). Investigation of blended cement hydration by isothermal calorimetry and thermal analysis. Cem. Concr. Res..

[48] Monteagudo S.M., Moragues A., Gálvez J.C., Casati M.J., Reyes E. (2014). The degree of hydration assessment of blended cement pastes by differential thermal and thermogravimetric analysis. Morphological evolution of the solid phases. Thermochim. Acta.

[49] Sun J., Tian L., Yu Z., Zhang Y., Li C., Hou G., Shen X. (2020). Studies on the size effects of nano-TiO_2_ on Portland cement hydration with different water to solid ratios. Constr. Build. Mater..

[50] Xu Z., Li W., Sun J., Hu Y., Xu K., Ma S., Shen X. (2017). Research on cement hydration and hardening with different alkanolamines. Constr. Build. Mater..

